# Risk Factors in Specialists and Generalists of Child-to-Parent Violence: Gender Differences and Predictors of Reactive and Proactive Reasons

**DOI:** 10.3390/bs13020085

**Published:** 2023-01-19

**Authors:** María J. Navas-Martínez, M. Carmen Cano-Lozano

**Affiliations:** Department of Psychology, University of Jaén, 23071 Jaén, Spain

**Keywords:** child-to-parent violence, reactive reason, proactive reason, specialist aggressors, generalist aggressors, gender differences, emotional intelligence, parenting practices

## Abstract

Recent research on child-to-parent violence (CPV) is advancing in the analysis of the specialist profile (aggressors who show only CPV) and the generalist profile (aggressors who show peer violence in addition to CPV). However, although differences have been found between girls and boys in the risk factors for CPV, there are no studies that analyze these differences according to the type of aggressor. Likewise, the importance of identifying the factors that differentially predict reactive and proactive CPV has been noted but has not been examined in different types of aggressors. The aims of this study were to examine gender differences in CPV patterns, emotional intelligence, parental victimization, and parental permissiveness and to analyze whether these variables predict reactive and proactive CPV, both according to aggressor type. A total of 1559 Spanish CPV aggressors (54.6% females) aged between 12 and 18 years from educational centers participated in the study (22.4% exercised only CPV (specialists) and 77.6% exercised peer violence in addition to CPV (generalists)). In general, no differences were found between girls and boys in the specialist profile, but differences were found in the generalist profile. Specifically, generalist girls exercised more psychological and control/domain violence toward mothers, while boys exercised more physical violence toward fathers and had more parental permissiveness. In specialists, parental victimization predicted reactive CPV, while parental permissiveness predicted proactive CPV. In contrast, in generalists, both parental victimization and parental permissiveness predicted both reactive and proactive CPV. Low emotional regulation was a significant predictor in both cases. This study identifies differences among girls and boys in CPV risk factors and among variables that predict reactive and proactive CPV and that these results differ between specialist and generalist aggressors. Implications for research and professional practice are discussed, highlighting the need to design and implement prevention and intervention programs specialized in the type of aggressor, paying special attention to gender differences and to the factors that motivate one or the other type of CPV.

## 1. Introduction

In recent years, one of the social problems of greatest concern is violence by sons and daughters toward their parents, or child-to-parent violence (hereinafter CPV). This type of family violence was first identified in 1957 under the name “battered parent syndrome” [1]. Since then, numerous studies have shown its presence in several countries [2,3,4,5].

One of the most accepted definitions by the scientific community conceptualizes CPV as “any act of a child that is intended to cause physical, psychological or financial damage to gain power and control over a parent” [6] (p. 3). In addition, the intention to obtain domain over parents is also considered a central aspect of CPV [7]. Likewise, the constitutive behaviors of CPV include three characteristics, which are reiteration, conscience, and intentionality [8]. Therefore, isolated or occasional behaviors, those that can occur under the effects of toxic substances or abstinence syndromes, and those that can be explained by the presence of psychological or developmental disorders are excluded [9].

Research on CPV has received progressive attention mainly due to its high prevalence in different countries, for example, in North and South America [10,11,12] or Europe [5,13,14]. More specifically, in these studies conducted with adolescents aged between 12 and 18 years, rates of verbal and psychological violence ranged from 15.7 to 80%, physical violence from 2.1 to 25%, financial violence from 9.3 to 50.9%, and control and domain behaviors from 19.4 to 36.0%.

Several studies point out that CPV has a multicausal origin (see Simmons et al. [5], for review) and, consequently, most intervention programs focus on working at various levels (individual, family, and social; see Toole-Anstey et al. [15], for review). However, these programs provide a generalized response in the treatment of CPV, without considering that different types of aggressors would require specialized responses for each of them. In this sense, it has been pointed out that understanding different typologies of aggressors can have important implications for the design and implementation of effective intervention programs [16]. In this line, a part of the current research examines the differences among diverse profiles of adolescents involved in CPV. Specifically, the typologies that have been analyzed so far classify aggressors according to the victimization or violence received [17], the childhood adversity experienced [18], the severity of the violence exercised [19,20], the generality of criminal behavior [21,22], and the generality of violent behavior, either towards other family members or outside the family [23,24,25]. The findings of these studies show the convenience of adapting interventions to the specific needs that the aggressors of each typology present.

More specifically, the profiles that are receiving most attention are those related to the influential dimension of the generality of criminal/violent behavior proposed by Holtzworth-Munroe and Stuart [26] in the field of gender-based violence. This dimension identifies two subtypes of aggressors according to their involvement only in family violence (family-only; hereinafter specialist aggressors) or in criminal behavior and extrafamilial violence in addition to family violence (generally antisocial/violent; hereinafter generalist aggressors). In CPV, Moulds et al. [22] found that 14.8% of offenders committed crimes related only to CPV (specialist CPV offenders), while 85.2% committed crimes related to CPV and also other types of crimes (generalist CPV offenders). In addition, 75% of generalist CPV offenders committed other crimes of a violent nature compared to 25% who committed crimes of a non-violent nature [22]. These authors conclude that CPV tends to occur within the context of a behavioral pattern of generalized violence, which is consistent with the figures found by Navas-Martínez and Cano-Lozano [24] in community adolescents. Specifically, 22.4% of the aggressors were classified as specialists, given that they exercised only CPV, whereas 77.6% were classified as generalists, given that they exercised both CPV and peer violence.

The above figures suggest that the majority of aggressors are involved in other forms of youth violence in addition to CPV. In this line, previous studies have found that adolescents who exercised CPV, compared with those who did not participate in this type of violence, carried out other types of youth violence to a greater extent [19,27], such as peer violence [19]. In the specific case of peer violence, it has been pointed out that it increases the risk of violence in general by approximately two-thirds (see Ttofi et al. [28], for review) and of CPV in particular [2,29]. Thus, the involvement of adolescents in peer violence may increase the risk of CPV and vice versa, being necessary to break this cycle of generalized violence. However, the figures referring to specialist aggressors also suggest that at least a part of adolescents who exercise CPV represent a pure group that would be worth examining in order to better understand how CPV behaves in isolation from other forms of violence.

Despite the previously mentioned findings, understanding of the typology of specialist and generalist aggressors is still very limited, given that only one study has examined it [24]. This study found that generalists exercised more CPV (psychological, physical, and financial) toward mothers and fathers and more control and domain behaviors toward mothers than specialists. Also, generalist aggressors provided more reactive or defensive and proactive or instrumental reasons for exercising CPV compared to specialist aggressors. Regarding the characteristics of these aggressors, Navas-Martínez and Cano-Lozano [24] found more difficulties in recognizing, assimilating, and regulating one’s own emotions in generalists than in specialists. Also, generalists experienced significantly higher levels of violence by their parents and had more permissive parental discipline than specialists. These results suggest a more negative profile in generalist aggressors than in specialists on these variables. However, although the differences between these two types of aggressors have already been analyzed in the aforementioned variables, gender differences in these variables have still not been analyzed.

Analyzing the role of gender in CPV could be important to improving the efficiency of interventions since areas that could be more problematic in girls than in boys involved in CPV and vice versa have been identified in the literature. In this regard, it would be worth examining whether the variables that differentiate specialist girls and boys are the same or different from the variables that differentiate generalist girls and boys. For example, some community studies found that girls exercise more psychological CPV [14,30,31] and boys exercise more physical CPV [30,31], although other studies found no differences [10,12,32]. Regarding CPV motivation, in general, it is found that girls inform more reactive reasons than boys, even though no differences are found in proactive reasons [17,33]. In the same line, lower levels of emotional intelligence have been found in boys than in girls [34]. In contrast, in judicial samples, higher levels of emotional instability are found in girls than in boys [35]. When the different dimensions of emotional intelligence were analyzed, it was found that boys have more difficulty recognizing the emotions of others and girls have more difficulty in a greater number of emotional competences, in particular in the recognition and regulation of their own emotions [17]. Likewise, girls report more parental victimization than boys [36,37], although other studies do not find differences either in this type of parental practice or in others such as parental permissiveness [12,38,39]. The analysis of profiles and, in turn, according to gender could bring further clarity to some of the inconsistencies found in the literature on gender differences in CPV.

Another aspect of interest is adolescents’ reasons for exerting CPV. Traditionally, two types have been identified [40]; CPV motivated by reactive reasons occurs as a response to a perceived threat of harm, and CPV motivated by proactive reasons occurs as an instrument to obtain self-benefits. Despite its relevance, few studies have explored its role [3,17,21,33,41,42,43], and only one has done so regarding the specialist and generalist profile [24], with further research being necessary. In this line, the importance of identifying which specific factors predict reactive and proactive CPV has been pointed out since different factors seem to trigger different types of CPV. For example, it was found that family victimization is related to reactive CPV through some components of social information processing such as anger, and to proactive CPV through other components such as justification of violence [21,42,43]. However, there are no studies on profiles that have explored whether the factors that explain reactive CPV are different from the factors that explain proactive CPV and whether they differ according to the type of aggressor. Further research in this direction could be useful in focusing interventions on the type of violence used by each type of aggressor based on the specific factors that trigger them. In this line, one theory suggests that specialist aggressors would exercise reactive CPV in response to harsh parental discipline in terms of parental authority and violence, whereas generalist aggressors would exercise proactive CPV due to the influence of permissive parental discipline [44]. However, it was found that generalist aggressors, while they informed more proactive reasons for exerting CPV and had more permissive parental discipline than specialists, also informed more reactive reasons and experienced more parental violence than specialists [24]. Thus, the available evidence suggests that reactive CPV and parental victimization would not be more characteristic of specialists and that proactive CPV and parental permissiveness would not be the only variables more characteristic of generalists.

Based on the literature review this study aimed to deepen the understanding of the profile of adolescents who have shown violent behaviors toward parents (specialists) and those who have shown violent behaviors toward parents and peers (generalists). To this end, two aims were set. The first was to analyze gender differences in the pattern of CPV (types of violence and types of reasons), in emotional competences (intrapersonal and interpersonal emotional perception, emotional assimilation, and emotional regulation), and in two types of parental practices (parental victimization and parental permissiveness), and whether they differ according to the type of aggressor. The second aim was to examine the variables that predict reactive CPV and proactive CPV and whether they differ according to the type of aggressor. No hypotheses are established given the lack of previous studies that have analyzed these questions.

## 2. Materials and Methods

### 2.1. Participants

Applying a non-probabilistic intentional sampling method, adolescents from 14 educational centers located in two provinces of southern Spain were selected. The initial sample consisted of 3142 adolescents, and from these, those who showed repeated CPV behaviors during the last year (any behavior exercised 2 or more times) were selected. The final sample consisted of 1559 CPV aggressors (54.6% female) aged between 12 and 18 years (*M*_age_ = 14.5, *SD* = 1.5) of Spanish nationality (98.1%) from public (50.5%) and charter schools (49.5%). A total of 75.3% live with both parents and the rest come from single-parent or restructured families in which the adolescent lives together with one of the two parents. The majority indicate that they are their parents’ biological children (98.3%) and that their parents are married (73.5%). A total of 22.4% of the 1559 CPV aggressors did not exercise violence towards peers (*n* = 349, specialist aggressors), while 77.6% did exercise violence towards peers (*n* = 1210, generalist aggressors).

### 2.2. Tools

Child-to-parent violence: Child-to-Parent Violence Questionnaire, Adolescent version (*CPV-Q* [45]). The questionnaire assesses the frequency with which violent behaviors (psychological, physical, financial, and control/domain) are exercised toward the mother (α = 0.67) and father (α = 0.66) during the last year through 14 parallel items scored on a Likert scale (0 = never; 4 = very often, six times or more). The second part of the questionnaire assesses adolescents’ reasons for exercising violence toward the mother (reactive: α = 0.61; proactive: α = 0.66) and the father (reactive: α = 0.60; proactive: α = 0.66) through 8 parallel items scored on a Likert scale (0 = never; 3 = always).

Peer violence: European Bullying Intervention Project Questionnaire and European Cyberbullying Intervention Project Questionnaire (*EBIP-Q* and *ECIP-Q* [46], Spanish validation [47]). Both instruments assess the frequency with which violent behaviors toward peers are exercised directly (bullying: α = 0.74) and by digital devices (cyberbullying: α = 0.68) in the last two months through 7 and 11 items, respectively, scored on a Likert scale (0 = no; 4 = yes, more than once a week).

Emotional intelligence: Wong and Law Emotional Intelligence Scale (*WLEIS* [48], Spanish validation [49]). This assesses the global degree of emotional intelligence through 16 items scored on a Likert scale (0 = completely disagree; 7 = completely agree) and four dimensions of the construct: intrapersonal emotional perception, or valuation and expression of one’s own emotions (α = 0.75); interpersonal emotional perception, or valuation and recognition of others’ emotions (α = 0.68); emotional assimilation, or use of emotions to facilitate personal performance (α = 0.78); and emotional regulation, or ability to manage one’s own emotions (α = 0.81).

Parental victimization: Violence Exposure Scale (*VES* [41]). This assesses the frequency with which violent behaviors (psychological, physical, verbal) are experienced by parents (α = 0.87) through 6 items scored on a Likert scale (0 = never; 4 = every day).

Parental permissiveness: Attachment Representations Questionnaire, short version (*CAMIR-R* [50], Spanish validation [51]). One of the dimensions of this instrument assesses the adolescent’s perception of the use of permissive discipline by their parents (α = 0.60) through 3 items scored on a Likert scale (1 = strongly disagree; 4 = strongly agree).

### 2.3. Procedure

This cross-sectional research presents a descriptive (ex post facto) design of populations using surveys [52]. The study was conducted according to the guidelines of the Declaration of Helsinki and approved by the Ethics Committee of the University of Jaén (MAR.18/5.PRY). Subsequently, authorization was obtained from both the public administration in the field of education and all educational centers. Next, the parents received written information about the study, and their signed informed consent was obtained. Following that, the authorized adolescents were informed and their signed informed consent also was obtained. The participation of these adolescents consisted of voluntarily completing a set of completely anonymous and confidential paper-and-pencil questionnaires. The administration of the questionnaires was carried out by a single evaluator in person and in groups in the classrooms of the educational centers. It was confirmed with the information provided by the educational centers that the participants included in this study did not have psychological or developmental disorders or special educational needs at the time of data collection. The classification criteria for participants in both study groups are based on self-reported violence (e.g., [19,20,32,53]). The “specialist group” included CPV aggressors who exercised neither bullying (any behavior 0 times) nor cyberbullying (any behavior 0 times) in the last two months. More specifically, these aggressors report exercising all bullying behaviors and all cyberbullying behaviors with zero frequency (0 = never). The “generalist group” included CPV aggressors who exercised bullying (any behavior 1 or more times) or cyberbullying (any behavior 1 or more times) in the last two months.

### 2.4. Data Analysis

Cronbach’s α was used to assess the internal consistency. The α value for all statistical tests was set to 0.05. Differences according to aggressor gender in the independent variables (CPV pattern, emotional competences, and parenting practices) were analyzed with the *t*-test for independent samples. The effect sizes of the differences were calculated with Cohen’s *d* statistic test (*d* = 0.20 (small), 0.50 (medium), 0.80 (large)) [54].

Following the examination of the relationships between the study variables with correlational analyses, four multiple linear regression analyses were conducted for each of the two aggressor groups. In all regressions, the outcome or dependent variables were reactive (toward the mother and the father) and proactive (toward the mother and the father) CPV reasons. The predictor or independent variables were gender (1 = females), the four dimensions of emotional intelligence, and the two types of parenting practices. All assumptions of the regressions were met except the normality and homoscedasticity of the residuals. Despite this, some authors claim that these two assumptions could be violated in those cases in which the nature of the data is cross-sectional and the sample is larger than 30 participants, which would only imply a reduction in the predictive capacity of the model (e.g., [55]). Likewise, the analyses of this study are supported by other research on CPV using the same analytical strategy in non-normal distributions (e.g., [2]).

## 3. Results

The pattern of CPV does not differ between girls and boys in the specialist group, except for the reasons for CPV (see Table 1). Specifically, girls obtain higher scores on reactive CPV toward mothers than boys (*F* (344.14) = 4.98, *p* = 0.026, *d* = 0.24). In the generalist group, girls present higher scores on psychological violence (*F* (1203) = 2.37, *p* = 0.014, *d* = 0.14) and on control and domain behaviors toward mothers (*F* (1189.80) = 2.83, *p* = 0.004, *d* = 0.17), while boys obtain higher scores on physical violence toward fathers (*F* (1191) = 22.72, *p* = 0.013, *d* = −0.14). Generalist girls obtain higher scores on reactive CPV toward mothers (*F* (1203) = 17.02, *p* < 0.001, *d* = 0.39) and fathers (*F* (1191) = 10.42, *p* < 0.000, *d* = 0.34) than boys from the same group.

The results show statistically significant gender differences in the specialist group in emotional competences (see Table 2). Specifically, girls obtain lower scores in the ability to recognize (*F* (346) = 11.08, *p* < 0.001, *d* = −0.49) and to regulate one’s own emotions (*F* (338.96) = 0.45, *p* = 0.019, *d* = −0.25), while boys obtain lower scores in the ability to recognize others’ emotions (*F* (343.79) = 0.80, *p* = 0.006, *d* = −0.30). In the generalist group, the results show statistically significant gender differences in both emotional competences and parenting practices. In particular, girls obtain lower scores in the ability to recognize one’s own emotions (*F* (1208) = 7.22, *p* < 0.001, *d* = −0.33) and to regulate them (*F* (1163.35) = 0.19, *p* < 0.001, *d* = −0.26), while boys obtain lower scores in the ability to recognize others’ emotions (*F* (1208) = 5.01, *p* < 0.001, *d* = 0.30) and higher scores in parental permissiveness (*F* (1177.70) = 0.48, *p* = 0.005, *d* = −0.16).

The correlational analysis (see Table 3) shows that female gender is positively related to reactive CPV toward the mother in both the specialist and generalist groups, and toward the father only in the generalist group. In both groups, reactive CPV is negatively related to intrapersonal emotional perception, assimilation, and emotional regulation and positively related to parental victimization. Only in the generalist group, reactive CPV is also positively related to parental permissiveness. Regarding proactive CPV, in the specialist group, it is negatively related to emotional regulation and positively related to parental permissiveness, whereas in the generalist group it is negatively related to all emotional intelligence dimensions and positively related to parental permissiveness and parental victimization.

The regressions show that while all other variables remain constant, specialist girls exercise more reactive CPV toward mothers than boys from the same group (see Table 4). On the other hand, generalist girls also exercise more reactive CPV toward mothers and fathers than boys from the same group (see Table 5).

In the specialist group (see Table 4), low levels of emotional regulation and high levels of parental victimization significantly predict reactive CPV toward mothers (18.9%; *F* (5, 345) = 15.87, *p* < 0.001) and fathers (13.7%; *F* (4, 346) = 13.60, *p* < 0.001). Low emotional regulation is a more relevant predictor of reactive CPV toward fathers, whereas high parental victimization is a more relevant predictor of reactive CPV toward mothers. In the generalist group (see Table 5), low levels of emotional regulation and high levels of parental victimization also significantly predict reactive CPV toward mothers (25.2%; *F* (6, 1193) = 66.72, *p* < 0.001) and fathers (25.2%; *F* (6, 1191) = 66.38, *p* < 0.001). However, in these cases, parental victimization is a more relevant predictor than emotional regulation in explaining reactive CPV toward both mothers and fathers and, in addition, parental permissiveness is also a significant predictor of this type of CPV toward mothers.

Finally, in the specialist group (see Table 4), low levels of emotional regulation and high levels of parental permissiveness significantly predict proactive CPV toward mothers (5.9%; *F* (2, 346) = 10.71, *p* < 0.001) and fathers (4.1%; *F* (2, 345) = 7.30, *p* = 0. 001), whereas in the generalist group (see Table 5), low levels of emotional regulation, high levels of parental permissiveness, and also high levels of parental victimization significantly predict this type of CPV toward mothers (6.1%; *F* (5, 1181) = 15.30, *p* < 0.001) and fathers (6.2%; *F* (5, 1179) = 86.69, *p* < 0.001). Low capacity to perceive others’ emotions also significantly predicted proactive CPV toward fathers.

## 4. Discussion

The first aim of this study was to analyze gender differences in the pattern of CPV, in emotional competences, and two types of parenting practices and whether they differ according to the type of aggressor. The results show that, in general, while specialist girls and boys share a similar profile, generalist girls and boys have a more different profile.

Specifically, regarding the CPV pattern, this study finds similar levels of CPV among specialist girls and boys. In contrast, in the generalist aggressors, while girls exercised more psychological violence and more control and domain behaviors toward mothers, boys exercised more physical violence toward fathers. In this sense, the lack of differences in CPV levels as a function of the specialists’ gender is consistent with community studies that also find no gender differences among CPV aggressors in general [10,12,32]. In contrast, the presence of gender differences among generalists is consistent with the results of other community studies where girls exercised more psychological violence [14,30,31] and boys more physical violence [30,31], and also with those in which psychological violence and control and domain behaviors are more directed toward mothers and physical violence toward fathers [10,11,56]. It has previously been noted that specialist CPV aggressors exercise less CPV than generalists [24], and this study provides that such violence is exercised by girls and boys in this group in a similar proportion. This could suggest that gender would not be a factor influencing the frequency of CPV exerted by specialist aggressors, although it could influence the frequency of CPV exerted by generalist aggressors. Specifically, in the case of generalist aggressors, in addition to being the most violent group [24], this study found that the type of violence differs according to gender, with girls exercising the most subtle forms of violence and boys the most direct forms, and there is also a correspondence between the gender of the aggressor and the gender of the victim. These results could be due to differences in the gender socialization process regarding the use of different types of violence, which includes psychological violence as a behavior more typically associated with women and physical violence with men. In this sense, it is likely that gender socialization determines in both girls and boys not only the different types of violence exercised but also the parent towards which such violence is directed. Regarding the reasons for CPV, in this study specialist and generalist girls exercised more CPV motivated by reactive reasons than boys from the same group, while no differences were found in proactive reasons, which is consistent with previous studies that find differences only in reactive CPV, more frequent in girls than in boys (e.g., [17,33]). Our findings support previous results that underline the reactive nature of CPV exercised by girls providing a novelty that these results are maintained even in different typologies of CPV aggressors.

With respect to emotional intelligence, the results of this study show that specialist and generalist girls had greater difficulties in recognizing and regulating their emotions than boys from the same group, while specialist and generalist boys had greater difficulties only in recognizing other people’s emotions than girls from the same group. Previous studies found greater emotional instability in girls who exercise CPV than in boys in community [17] and judicial samples [35], as well as higher levels of anger in girls with CPV crime compared to boys with this crime and even compared to girls and boys with other different crimes [21]. As in previous studies, our findings suggest that in general girls have more emotional difficulties than boys, results that are also confirmed in the different types of aggressors analyzed, providing new data. One explanation for these results would have to do with what has been found in previous studies that some emotional variables such as anger are specifically related to CPV motivated by reactive reasons [21,43]. Specifically, it may be possible that emotional difficulties related to self-regulation, which are more characteristic of girls than boys in this study, are largely responsible for reactive CPV, also more characteristic of girls than boys. These results could suggest the convenience not only of analyzing different types of aggressors but also in terms of gender.

Concerning parental practices, previous research has highlighted the relevance of parental victimization in CPV (e.g., [2,12,17,21,37,38,39,43,57]), increasing the probability of this type of violence in victimized adolescents by more than 70% [58]. More specifically, the profile research shows that generalist aggressors in particular reported significantly higher levels of parental victimization compared to specialist aggressors [24]. This study finds novel results showing that this variable is present in both girls and boys of both typologies, with no gender differences in the levels of victimization experienced. Based on our results, it would be useful for future studies to examine in different typologies of CPV aggressors, the differences between girls and boys in the type of violence suffered by their parents, also distinguishing the gender of the parent who exerted the violence. Finally, although it has previously been noted that generalist aggressors have more permissive parental discipline than specialists [24], this study finds that it is mainly the generalist boys who have parental permissiveness, more than generalist girls. Moreover, this is a specific characteristic of boys in this typology of aggressors, given that no gender differences are found in this variable in specialist aggressors. Wilson [59] suggested that when parents are too permissive there could be a change of roles that would imply that children take on the role of parents, which could generate conflicts that could lead to CPV. According to this idea and based on our findings, boys could take on this role of authority more than girls, which they could extrapolate to the school context, committing violent acts more likely in this context as well. This would be in line with the definition of school violence, according to which violence is exercised toward someone in a position of less power [60].

The second aim of this study was to examine the variables that predict reactive CPV and proactive CPV and whether they differ according to the type of aggressor. In terms of gender, the results confirm that girls, both specialists and generalists, would be more likely to use CPV for reactive purposes compared to boys from the same group. Overall, the findings of this study show that the parental variables predicting reactive CPV and proactive CPV are different in the case of specialist aggressors while they are the same in the case of generalist aggressors. Particularly, in specialist aggressors, while parental victimization specifically predicted reactive CPV, parental permissiveness specifically predicted proactive CPV. In contrast, in generalist aggressors, both parental victimization and parental permissiveness were significant predictors of both reactive and proactive CPV. In line with previous research, the findings of this study suggest that different risk factors lead to different types of CPV [21,42,43] and add further information by finding that these results differ among different typologies of aggressors. This highlights the need for further research in this direction. On the other hand, the study also shows that the emotional variables predicting reactive CPV and proactive CPV are the same for both specialist and generalist aggressors. Specifically, emotional regulation was the only dimension of emotional intelligence that contributed to predicting the types of CPV analyzed and in both types of aggressors. More specifically, a low ability to regulate emotions significantly predicted CPV motivated by reactive reasons and by proactive reasons. Although in line with previous research [21,43], in this study, low emotional regulation was more relevant in predicting reactive CPV than proactive CPV. These results underline the capacity of emotional dysregulation to trigger proactive and especially reactive CPV in these two aggressor types.

Based on the above, it seems that in this study it is the parental practices that make the difference between the type of CPV exercised by specialists and generalists, although not in line with what was proposed by Kuay et al. [44]. According to the model of these authors, specialists would exercise reactive CPV due to the influence of authoritarian and violent discipline, while generalists would exercise proactive CPV due to the influence of permissive discipline. Our results suggest that this model could explain specialized CPV but not generalized CPV. This is so given that the results show that specialists seem to specifically trigger reactive CPV in response to experienced parental victimization to defend themselves from that victimization, while these aggressors also seem to specifically trigger proactive CPV to obtain the benefits they are habituated to in the context of a permissive discipline. In these cases, denying them something they want may trigger violence [59]. In the adolescents exercising generalized violence, both victimization and permissiveness predicted both reactive and proactive use of CPV. Thus, contrary to what occurs in specialist aggressors, in generalist aggressors, the type of parental practice does not seem to discriminate the type of motivation to exercise CPV, it being likely that in these cases a combination of these factors and others linked to more generalized violence may do so.

In summary, in general terms, this study finds that specialist girls and boys present a similar profile and that the type of CPV they perform seems to depend on the type of parental practice received. Specifically, the pattern of CPV is similar between girls and boys in this group and they also do not differ in the parental practices analyzed, with parental victimization being a specific predictor of reactive CPV and parental permissiveness a specific predictor of proactive CPV. In contrast, generalist girls and boys, in general, present a different profile, and the type of CPV they perform does not seem to depend exclusively on a specific type of parental practice. Specifically, girls exercise more psychological violence and more control and domain behaviors toward mothers while boys exercise more physical violence toward fathers and have a more permissive parental discipline. In these cases, both victimization and parental permissiveness predict both reactive and proactive CPV. Finally, the results also show that girls in both groups exercise CPV for more reactive reasons and have more difficulty recognizing and regulating the emotions they experience, while boys in both groups have more difficulty recognizing the emotions of the people around them, and that low emotional regulation is a significant predictor of both types of reasons and in both types of aggressors.

This study presents some limitations that must be considered in the interpretation of the results. First, given the cross-sectional nature of the data, it is not possible to establish causal inferences. Longitudinal studies are necessary to evaluate the temporal sequence of the variables analyzed. Second, the sample belongs to a specific geographical and cultural context, which limits the generalizability of the results to other contexts. It would be convenient to replicate the results in populations from other provinces of Spain and other countries. Third, the data are obtained from adolescent self-reports, and it would be convenient to have additional data from other sources such as parents and/or peers.

Despite these limitations, this is the first study on CPV that analyzes gender differences and risk factors that predict reactive and proactive CPV in specialist and in generalist aggressors, providing relevant information for research and professional practice. At the research level, we suggest the need for future studies to analyze CPV by distinguishing aggressors according to different typologies and, in turn, according to gender. CPV aggressors may not be a homogeneous group, as recent evidence together with the results of this study show that CPV levels and their associated risk factors differ between different typologies of aggressors and even between girls and boys of each typology. Judicial studies conducted in Spain have found that CPV offenders differ in some risk factors depending on whether they are specialists or generalists [21] and depending on whether they are girls or boys [37]. Now, it would be interesting to analyze the differences in these risk factors as a function of the type of CPV offender and gender. In addition, it would be convenient for future studies to analyze whether different typologies of aggressors differ according to other sociodemographic variables such as age. On the other hand, more studies are needed to confirm the results on the capacity of the parental practices analyzed to differentially predict CPV motivated by reactive and proactive reasons in specialist aggressors. It would also be necessary to include in these models other types of predictors, such as anger, which has been associated with reactive CPV, or justification of violence, which is more associated with proactive CPV (e.g., [43]), to determine whether these results remain the same in specialist and generalist aggressors or whether they are more characteristic of one type than the other. Finally, future studies should take into account the moderating role of gender, and also age, in the relationship between CPV and associated risk factors.

On a practical level, it would be convenient for professionals to determine before the intervention the degree of generality of the violence through a record of the adolescent’s involvement in different types of youth violence in order to better orient the treatment. In this sense, it is suggested that interventions could emphasize working more actively with girls on the subtle forms of CPV and with boys on the more direct forms. Likewise, helping families and schools to identify CPV behaviors could be useful in preventing their reiteration, paying special attention to psychological violence and control/domain behaviors in girls and physical violence in boys. On the other hand, it has been pointed out that interventions that promote emotional and social skills reduce violent behaviors in adolescence (e.g., [61]). This study suggests the need to work on the emotional skills of all adolescents involved in CPV, regardless of their involvement in other types of youth violence, focusing on promoting intrapersonal skills or those skills related to dysregulation in girls and interpersonal skills or those skills related to empathy in boys. Finally, the results of the study might suggest that treatment of CPV in adolescents not involved in other forms of youth violence could include interventions more centered on bidirectional family violence history and violent relationship patterns when CPV is more reactive than proactive and interventions more centered on permissive parenting practices when CPV is more proactive than reactive. In contrast, in adolescents involved in other types of youth violence in addition to CPV, it would be appropriate for interventions to work on these aspects in equal measure regardless of the type of CPV.

## 5. Conclusions

In conclusion, although further research is needed, this study identifies differences among girls and boys in CPV risk factors and among variables that predict reactive and proactive CPV, showing additionally that these results differ between specialist and generalist aggressors. We suggest the need to design and implement specialized prevention and intervention programs according to the type of aggressor, paying special attention to gender differences and to the factors that motivate one or the other type of CPV.

## Figures and Tables

**Table 1:** Gender differences in child-to-parent violence pattern by aggressor type.

	Specialists					Generalists				
	Females *n* = 188		Males *n* = 160		*t*	Females *n* = 663		Males *n* = 547		*t*
	*M*	*SD*	*M*	*SD*		*M*	*SD*	*M*	*SD*	
CPV Mother										
Psychological	0.95	1.84	1.14	2.18	−0.92	2.64	3.01	2.22	2.80	2.46 *
Physical	0.09	0.44	0.07	0.41	0.44	0.21	0.81	0.26	0.98	−0.98
Financial	0.42	1.10	0.28	0.65	1.55	0.95	1.31	0.85	1.37	1.34
Control	2.95	1.93	2.79	2.03	0.75	3.56	2.44	3.16	2.25	2.88 **
Reactive	1.37	1.81	0.97	1.45	2.24 *	2.32	2.08	1.57	1.70	6.87 ***
Proactive	1.89	2.08	1.74	1.85	0.68	3.25	2.58	3.11	2.58	0.92
CPV Father										
Psychological	0.94	1.54	1.11	2.10	−0.82	2.42	2.91	2.15	2.86	1.58
Physical	0.05	0.25	0.04	0.23	0.36	0.15	0.63	0.28	1.15	−2.36 *
Financial	0.40	1.08	0.24	0.64	1.66	0.81	1.22	0.72	1.28	1.25
Control	2.53	1.77	2.44	1.87	0.45	2.88	2.36	2.70	2.13	1.39
Reactive	1.21	1.56	0.94	1.33	1.71	2.22	2.07	1.57	1.75	5.87 ***
Proactive	1.52	1.88	1.55	1.74	−0.16	2.62	2.46	2.63	2.45	−0.07

Note: CPV = child-to-parent violence; * *p* < 0.05; ** *p* < 0.01; *** *p* < 0.001.

**Table 2:** Gender differences in emotional intelligence and parenting practices by aggressor type.

	Specialists					Generalists				
	Females *n* = 188		Males *n* = 160		*t*	Females *n* = 663		Males *n* = 547		*t*
	*M*	*SD*	*M*	*SD*		*M*	*SD*	*M*	*SD*	
Emotional intelligence										
Intrapersonal	20.27	5.12	22.53	3.99	−4.64 ***	19.36	4.73	20.85	4.28	−5.70 ***
Interpersonal	22.13	3.95	21.00	3.64	2.77 **	21.81	3.86	20.44	4.22	5.92 ***
Assimilation	19.61	5.22	20.26	5.37	−1.15	18.64	5.30	18.97	5.27	−1.11
Regulation	18.16	5.25	19.48	5.16	−2.36 *	15.32	5.42	16.72	5.44	−4.47 ***
Parenting practices										
Victimization	2.61	3.80	3.05	4.23	−1.03	4.96	5.20	5.14	5.02	−0.60
Permissiveness	6.59	2.26	6.83	1.91	−1.07	6.92	2.28	7.28	2.21	−2.83 **

Note: * *p* < 0.05; ** *p* < 0.01; *** *p* < 0.001.

**Table 3:** Inter-correlations between the study variables.

	1	2	3	4	5	6	7	8	9	10	11
Gender	-	0.19 ***	0.16 ***	0.03	−0.00	−0.16 ***	0.17 ***	−0.03	−0.13 ***	−0.02	−0.08 **
CMV-Reactive	0.12 *	-	0.77 ***	0.22 ***	0.20 ***	−0.15 ***	0.03	−0.18 ***	−0.26 ***	0.42 ***	0.07 *
CFV-Reactive	0.09	0.75 ***	-	0.17 ***	0.24 ***	−0.14 ***	0.05	−0.15 ***	−0.25 ***	0.44 ***	0.06 *
CMV-Proactive	0.04	0.05	0.06	-	0.81 ***	−0.09 **	−0.03	−0.09 **	−0.15 ***	0.19 ***	0.11 ***
CFV-Proactive	−0.01	0.01	0.08	0.89 ***	-	−0.07 *	−0.07 *	−0.05	−0.13 ***	0.21 ***	0.10 ***
Intrapersonal	−0.24 ***	−0.18 ***	−0.15 **	−0.10	−0.09	-	0.21 ***	0.39 ***	0.49 ***	−0.09 ***	−0.02
Interpersonal	0.15 **	0.06	0.10	0.03	−0.05	0.25 ***	-	0.22 ***	0.16 ***	.03	−0.01
Assimilation	−0.06	−0.21 ***	−0.16 **	−0.02	−0.00	0.44 ***	0.28 ***	-	0.37 ***	−0.17 ***	−0.06 *
Regulation	−0.13 *	−0.32 ***	−0.31 ***	−0.20 ***	−0.17 **	0.52 ***	0.19 ***	0.32 ***	-	−0.17 ***	−0.02
Victimization	−0.05	0.33 ***	0.26 ***	0.00	−0.00	−0.10	−0.00	−0.23 ***	−0.19 ***	-	0.05
Permissiveness	−0.06	0.03	0.03	0.15 **	0.12 *	−0.12 *	0.02	−0.10	−0.10	0.00	-

Note: Results for the specialist aggressor sample (*n* = 349) are shown below the diagonal and those for the generalist aggressor sample (*n* = 1210) are shown above the diagonal. Gender: 1 = females; CMV = child-to-mother violence; CFV = child-to-father violence; * *p* < 0.05; ** *p* < 0.01; *** *p* < 0.001.

**Table 4:** Multiple linear regressions of the prediction of reactive and proactive child-to-parent violence by specialist aggressors.

Reactive Child-to-Parent Violence						Proactive Child-to-Parent Violence						
Mother			Father			Mother			Father			
	*B*	*SE*	β	*B*	*SE*	β	*B*	*SE*	β	*B*	*SE*	β
Constant	2.34 ***	0.48		2.34 ***	0.40		2.31 ***	0.53		1.98 ***	0.49	
Gender	0.34 *	0.17	0.10	-	-	-	-	-	-	-	-	-
Intrapersonal	0.01	0.02	0.03	0.01	0.02	0.04	-	-	-	-	-	-
Interpersonal	-	-	-	-	-	-	-	-	-	-	-	-
Assimilation	−0.02	0.02	−0.07	−0.01	0.02	−0.04	-	-	-	-	-	-
Regulation	−0.08 ***	0.02	−0.24	−0.07 ***	0.02	−0.27	−0.07 ***	0.02	−0.19	−0.06 **	0.02	−0.16
Victimization	0.11 ***	0.02	0.27	0.07 ***	0.02	0.20	-	-	-	-	-	-
Permissiveness	-	-	-	-	-	-	0.13 *	0.05	0.13	0.09 *	0.05	0.10
*R* ^2^	0.189			0.137			0.059			0.041		
∆R ^2^	0.177			0.127			0.053			0.035		

Note: Gender: 1 = females. Only predictor variables with significant relationships with the dependent variables are included, where (-) indicates no relationship. * *p* < 0.05; ** *p* < 0.01; *** *p* < 0.001.

**Table 5:** Multiple linear regressions of the prediction of reactive and proactive child-to-parent violence by generalist aggressors.

	**Reactive Child-to-Parent Violence**						**Proactive Child-to-Parent Violence**						
	**Mother**			**Father**			**Mother**			**Father**			
	***B***	***SE***	β	***B***	***SE***	β	***B***	***SE***	β	***B***	***SE***	β	
Constant	1.55 ***	0.32		1.37 ***	0.32		3.01 ***	0.45		2.87 ***	0.49		
Gender	0.72 ***	0.10	0.18	0.62 ***	0.10	0.16	-	-	-	-	-	-	
Intrapersonal	0.01	0.01	0.02	0.00	0.01	0.01	−0.01	0.02	−0.02	0.00	0.02	0.00	
Interpersonal	-	-	-	-	-	-	-	-	-	−0.03 *	0.02	−0.06	
Assimilation	−0.02	0.01	−0.05	−0.01	0.01	−0.01	−0.01	0.02	−0.01	-	-	-	
Regulation	−0.06 ***	0.01	−0.16	−0.05 ***	0.01	−0.15	−0.05 **	0.02	−0.11	−0.04 *	0.01	−0.08	
Victimization	0.15 ***	0.01	0.39	0.16 ***	0.01	0.41	0.08 ***	0.01	0.16	0.09 ***	0.01	0.19	
Permissiveness	0.05 *	0.02	0.06	0.04	0.02	0.05	0.12 ***	0.03	0.10	0.09 **	0.03	0.08	
*R* ^2^	0.252			0.252			0.061			0.062			
∆R ^2^	0.248			0.248			0.057			0.058			

Note: Gender: 1 = females. Only predictor variables with significant relationships with the dependent variables are included, where (-) indicates no relationship. * *p* < 0.05; ** *p* < 0.01; *** *p* < 0.001.

## Data Availability

The data presented in this study are available on request from the corresponding author.
